# Field *et al*. Redux.

**DOI:** 10.1186/2041-9139-4-5

**Published:** 2013-02-12

**Authors:** Maximilian J Telford

**Affiliations:** 1 Department of Genetics, Evolution and Environment University College London, Darwin Building Gower Street, London, WC1E 6BT, UK

**Keywords:** Phylogeny, Evolution, Metazoa, SSU, Ribosomal RNA, Maximum likelihood

## Abstract

On 12 February 1988 (by coincidence Charles Darwin’s birthday), a paper published in *Science* by Katherine Field, Rudy Raff, and colleagues presented the first credible molecular analysis of metazoan phylogeny based on sequences from the small subunit ribosomal RNA gene (SSU). Here I examine the main conclusions reached in this manuscript. I reconstitute their dataset and, by recompiling software available in 1988, I consider how they might have achieved a more accurate tree. I show how three common methods to avoid systematic error - more data, careful taxon sampling and superior models of evolution - overcome the errors that exist in the original paper. This approach illustrates the basis of some of the major advances of the past 25 years resulting in our current understanding of animal phylogeny.

## Background

Twenty-five years ago, on the 179th anniversary of Charles Darwin’s birthday, a paper published in *Science* began a revolution in the study of animal phylogeny [1]. The paper reported the first use of small subunit ribosomal RNA sequences (SSU) for reconstructing animal phylogeny and adopted the technique for fast, direct sequencing of ribosomal RNA using reverse transcriptase pioneered by Norman Pace and others [2]. The work, from a team led by Rudy Raff, is referred to familiarly in the community as ‘Field *et al*.’ after the first author, Katherine Field (from here I refer to the paper as ‘FEA’). FEA set the scene for hundreds of subsequent SSU-based analyses of metazoan phylogeny; among these several equally influential papers that collectively have overturned traditional thinking about metazoan evolution resulting in what has been termed the ‘new animal phylogeny’ [3].

While hugely influential and widely cited, not all of the citing articles refer to FEA as a trustworthy guide to metazoan phylogeny. While globally accurate, there are some important errors and the critical response was almost immediate, with letters quickly appearing in the pages of *Science*[4-7]. The two most serious errors in their phylogeny are: (1) polyphyletic origins of Metazoa (independent origins of cnidarians and bilaterians); and (2) non-monophyletic Lophotrochozoa (Platyhelminthes grouped basal to other bilaterians rather than alongside molluscs, annelids, and relatives). Here I describe the reconstitution of their sequence alignments and I use this dataset to recapitulate the original analyses. I then consider whether it was, in principle, possible to produce a more accurate phylogeny with the resources available at the time of the original work. Finally I ask what subsequent advances in theory and technology were needed to arrive at the metazoan phylogeny generally accepted today.

### Main text

#### Reconstituting the original dataset

I downloaded the original FEA data files from GenBank. The majority of these are composed of three separate sequences from each taxon covering different subregions of the SSU molecule: these original data form dataset 1. To complement these partial sequences I added complete SSU sequences derived from all taxa represented by a partial sequence in the original dataset. Where a full sequence from the identical species was not available I used data from the closest relative I could find according to the GenBank taxonomy. I also added full length sequences from a slowly evolving platyhelminth to replace the rapidly evolving *Dugesia*: these form dataset 2. All sequences were aligned using Clustalx. I made character sets for inclusion of nucleotide positions in analyses corresponding to the positions used to reconstruct the trees shown in FEA-figs 2 to 5. I also made my own character set covering the whole SSU gene using the complete sequences. An additional file containing all aligned sequences, character sets used and a PAUP block to run all analyses described is available for download (see Additional file 1).

#### Phylogenetic analyses

**Figure 1:**
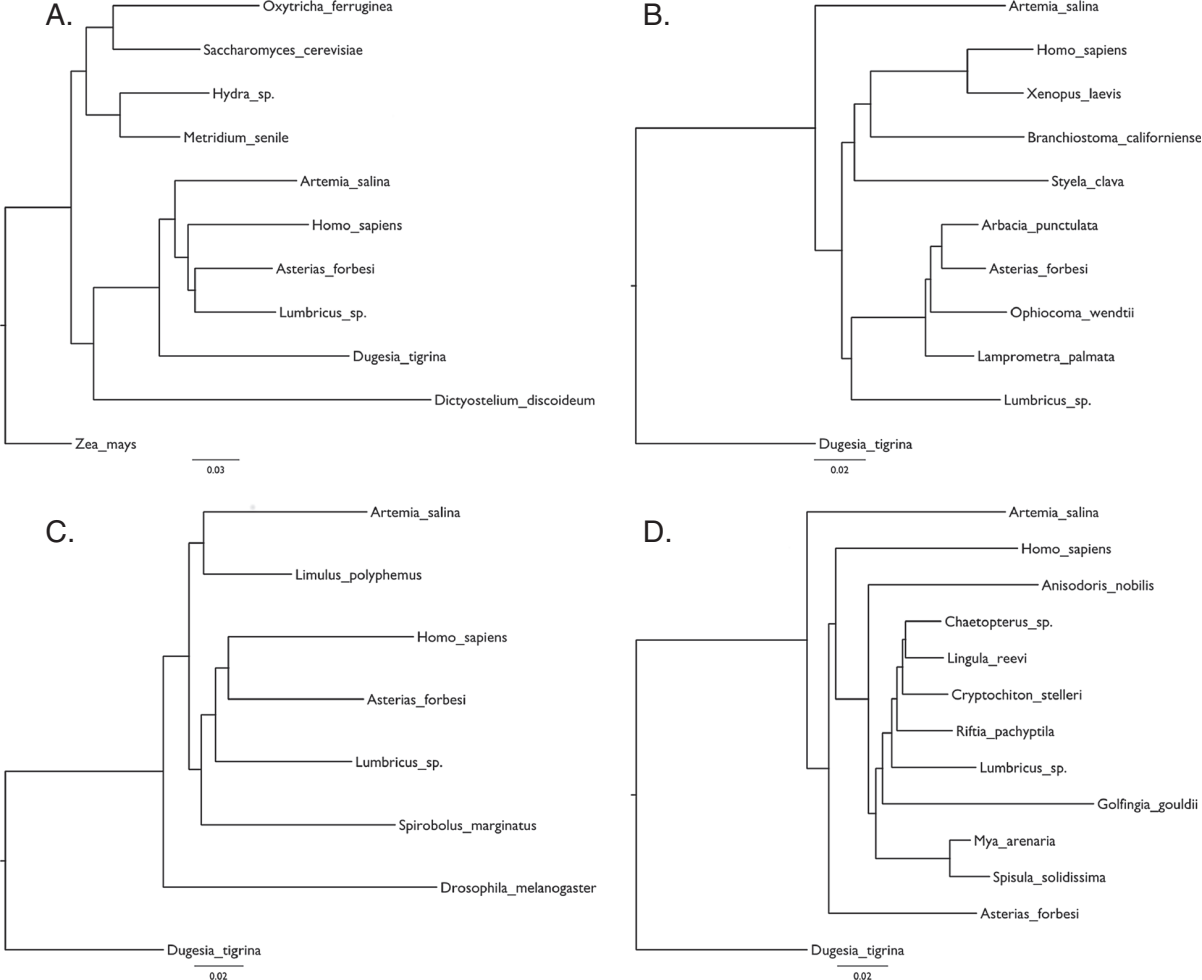
**Recapitulation of the analyses in Field *et al*. figures 2 to 5 using original data and methods of analysis.** (**A**) Recapitulation of FEA Figure 2 ‘Evolutionary tree for animals’. (**B**) Recapitulation of FEA Figure 3 ‘Chordate and echinoderm portions’. (**C**) Recapitulation of FEA Figure 4 ‘Arthropod portion’. (**D**) Recapitulation of FEA Figure 5 ‘Eucoelomate protostome portion’. Slight differences to the original results are discussed in the text.

**Figure 2:**
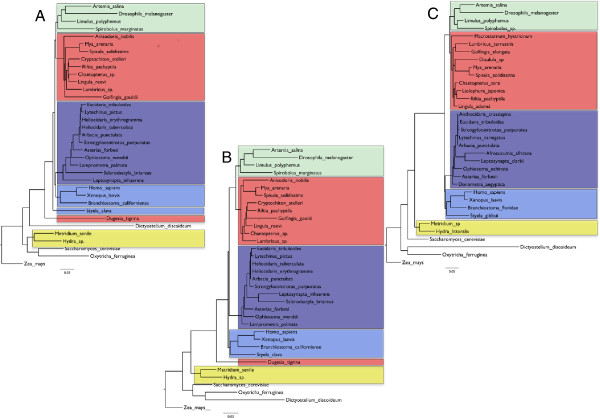
**Metazoan phylogenies using small subunit ribosomal RNA data.** (**A**) A recapitulation of the original Field *et al*. analysis using all original sequences in dataset 1. Three main errors are seen: (1) the cnidarians *Hydra* and *Metridium* (yellow) are separated from other Metazoans; (2) the flatworm *Dugesia* is separated from other Lophotrochozoans (red); and (3) the urochordate *Styela* is separate from other chordates (light blue). (**B**) Tree using the original data (dataset 1) re-analysed with a 1988 Maximum Likelihood method. Metazoa and chordates are now monophyletic. (**C**) Analysis of full length SSU sequences with long branched *Dugesia* replaced by shorter branched *Macrostomum* (dataset 2) and analysed with modern ML methods (GTR+I+G model). The flatworm is found with the other Lophotrochozoa. Arthropods are in green, echinoderms are in dark blue.

All phylogenetic analyses were done using PAUP 4.0b10 [8] and Phylip 3.0 [9]. Trees are rooted on *Zea mays* (maize) rather than *Dictyostelium discoideum* as in FEA, as the latter is now considered closer to the opisthokonts (Metazoa, fungi and others) than are plants or the ciliophoran *Oxytricha*[10]. I first re-ran the heuristic distance matrix-based analyses shown in FEA Figs 2 to 5 using the same taxa, the original sequences and the same portions of alignment (Figure 1) as well as an analysis using all the original taxa simultaneously using the character set from FEA Fig 2. Jukes Cantor distance estimates were derived from each set of partial sequences. Using PAUP, trees were compared using a heuristic search with a random addition of taxa (10 replicates) and tree bisection and reconnection (TBR) branch swapping. The optimality criterion was weighted least squares with power = 2. Using all sequences at once, a single best tree (Figure 2A) was found.

The analysis shown in Figure 2B used a Maximum Likelihood method performed using software available at the time of FEA. I downloaded and compiled DNAML from Phylip version 3.0: April 1987 [9]. The maximum number of species that could be considered by the software was originally fixed at 20 and the maximum number of characters was just 300 reflecting limitations of CPUs and available computer memory in 1988; both values were increased before compiling to accommodate the datasets used here. Maximum Likelihood estimates of base frequencies and transition: transversion ratios were based on the tree shown in Figure 1A and were estimated using PAUP 4.0b10 (Assumed nucleotide frequencies A= 0.25079 C= 0.23583 G= 0.24494 T= 0.26843, transition/transversion ratio = 1.4497). Global rearrangements G option was selected and the analysis was run 10 times using different random numbers with the Jumble J option and the tree with the highest ln-likelihood retained.

The analysis shown in Figure 2C used a Maximum Likelihood method from PAUP 4.0b10. Full-length sequences were used and the fast evolving flatworm *Dugesia* was replaced with the more slowly evolving *Macrosotomum* (dataset 2). An initial neighbour joining (NJ) tree was used to get a Maximum Likelihood estimate of nucleotide frequencies, the gamma parameter, the proportion of invariant sites and the GTR substitution matrix. These parameter estimates were fixed and the tree estimated using a heuristic search with a random addition of taxa (10 replicates) and TBR branch swapping. After this procedure, the parameters (GTR substitution matrix, base frequencies, gamma parameter and proportion of invariant sites) were re-estimated on the resulting tree and this procedure repeated until there was no further change in topology or estimates of parameters.

## Results and discussion

Rerunning the original analyses from FEA Figs 2–5 using a heuristic distance method gave similar but not identical results (Figure 1). In my recapitulation of FEA Fig 3 the arthropod *Artemia* is basal and the annelid *Lumbricus* sistergroup to the Echinoderms rather than the other way around as seen in the original. In my recapitulation of FEA Fig 4 the position of the root is different resulting in non-monophyletic arthropods. The differences may be due to improved methods for searching tree space possible today in PAUP* and the positions of the taxa that differ between FEA and my re-analyses seem to be particularly unstable. Figure 2A shows the tree reconstructed using all the partial sequences and tree reconstruction methods of the original paper. While FEA did not use all sequences in a single analysis, probably due to computational burden or memory requirements, this tree nevertheless recapitulates most of their findings well. Currently accepted major monophyletic groups are highlighted by shading: Chordata, dark blue; Echinodermata, light blue; Lophotrochozoa, red; Arthropoda, green; Cnidaria, yellow. The polyphyletic origins of the Metazoa are recovered as is the basal position of the platyhelminth *Dugesia tigrina*, distant from the other Lophotrochozoa. The long branched urochordate *Styela* is not close to the other chordates.

Alongside the major errors I have described, there are a number of additional errors within the major clades such as the basal position of the long-branched sea cucumbers (*Sclerodactyla briareus* and *Leptosynapta inhaerens*) within the echinoderms and paraphyly of molluscs (the bivalves *Mya* and *Spisula* are not sister to the chiton *Cryptochiton*).

One question of great historical interest asks how much improvement in the accuracy of the tree was theoretically possible at the time of publication, if optimal tree reconstruction methods had been used. Using the simple Maximum Likelihood (ML) models represented in software available at the time I have reconstructed the tree shown in Figure 2B. This likelihood approach produces a significant improvement in the tree topology, the Metazoa are now monophyletic; the sea cucumbers are no longer at the base of the echinoderms but in their correct position as sistergroup of the echinoids and the urochordate *Styela* has joined the other chordates suggesting that LBA is less of a problem. The major remaining error concerns the position of the platyhelminth, *Dugesia* basal to all other Bilateria.

Clearly there was the possibility in 1988 to approach closer to the tree widely accepted today; practically, however, maximum likelihood methods are hugely slower than the distance methods used in the original paper and computer CPUs are more than 10,000 times faster than in 1988 and the random access memory (RAM) available is orders of magnitude greater. Performing the 10 iterations used to produce the tree shown in Figure 2B took approximately 2 hours on my desktop computer yet would have taken at least 2 years in 1988. Even with 10 iterations I have not recovered the tree with the highest likelihood; running the same model using PAUP* produced a tree with a lnLikelihood of -10,287 (compared to -10,294 using PHYLIP 3.0).

The increase in accuracy of molecular phylogenetic trees of the past 25 years depends on three main developments in phylogenetics: systematic errors are reduced by longer sequences (now typically hundreds or even thousands of genes) and systematic errors are addressed by careful and more extensive species sampling and improved likelihood methods [11]. I have implemented all three approaches in a stepwise fashion. Using full-length SSU sequences results in subtle changes in the tree, some beneficial (brittlestar, *Ophiocoma*, in a more credible position) yet some detrimental (paraphyletic deuterostomes) suggesting that, while stochastic error might have reduced, systematic errors are still present. Replacing the rapidly evolving platyhelminth *Dugesia* by the shorter branched *Macrosotomum* clearly improves the tree, however, and results in the flatworm correctly joining the other lophotrochozoans. This approach is perhaps best exemplified by the work of Aguinaldo *et al*. [12] who found support for the Ecdysozoa (including nematodes and arthropods) only following selection of slowly evolving nematodes. Finally, the simple ML model implemented in the 1987 Phylip software models just two types of substitution (transitions and transversions) and has no parameters allowing for variation in substitution rates across sites. The use of a complex ML model incorporating a General Time Reversible model of substitution (all six possible substitutions modeled independently), an estimate of the proportion of invariant sites and a gamma model to account for variable rates across sites (GTR+I+G) results in a tree that closely approximates the most recent estimates of metazoan evolution (Figure 2C) [13-15]. Not only are chordates and lophotrochozoans now monophyletic but, within clades, we also see the close relationship of sea cucumbers with echinoids and monophyletic molluscs.

## Conclusions

It seems to have been through a mixture of pragmatism, good judgement and extraordinary good fortune that the 18S gene was chosen as the early workhorse for animal phylogenetics. While ribosomal RNA genes were used early on in phylogenetics due to the possibilities for extracting and purifying rRNA in large quantities, its amenability to direct sequencing and its universality, three additional features of 18S are the key to its subsequent successes. First, its length meant that stochastic or sampling errors were much reduced when compared to previous work on the 5S gene and in general it contains enough information to support the accurate estimation of the multiple parameters of the new models I have described. Second, it is composed of regions of greater and lesser sequence conservation meaning it has provided evidence for relationships at many different levels of the animal phylogeny and indeed across the whole of life. Finally, being a non-coding RNA means no problems from a degenerate code: the most tightly constrained regions of 18S hardly change meaning, come the PCR revolution, designing universal primers to amplify the gene from any animal was trivial. Compare this situation to the degenerate primers required to amplify a protein coding gene with neutrally variable first and third codon positions.

While I have studied the reasons behind the shortcomings of FEA, this truly was a landmark paper; a number of their conclusions were both revolutionary and correct and they did not by any means over interpret their results at the time. Two of their conclusions are of particular significance as they overturned entrenched ideas of animal evolution and their eventual acceptance forced a reassessment of the pattern and process of metazoan evolution.

(1) The brachiopods are more closely related to annelids and molluscs (Protostomia) than to the hemichordates (Deuterostomia) meaning the lophophore, that both these groups possess, is likely to be a convergent adaptation [16];

(2) The arthropods are the sister group of the other protostomes represented in the dataset (all Lophotrochozoa) and are not closely related to or derived from within the annelids (as per the Articulata hypothesis) raising questions about the evolution of body segmentation still hotly debated [12].

Both of these observations were counter to prevailing thought, each has since been demonstrated correct in high profile papers and each has revolutionised our understanding of how animals evolved [17]. The 25th anniversary of Field *et al*. deserves to be marked and its coincidence with Darwin’s birthday is both remarkable and fitting.

## Abbreviations

FEA: Field *et al*.; GTR+I+G: General Time Reversible, Invariant sites, Gamma correction; LBA: Long branch attraction; ML: Maximum Likelihood; NJ: Neighbour joining; SSU: Small subunit ribosomal RNA; TBR: Tree bisection and reconnection

## Competing interests

The author declares that he has no competing interests.

## Supplementary Material

Additional file 1 ** Telford_FEA.nex, Format: Nexus.** Description: Aligned 18S ribosomal RNA sequences from original Field *et al.* publication along with more recent, full-length, equivalent sequences. Character sets used in the analyses described are included along with a ‘PAUP block’ which will automatically run all analyses described in this manuscript. Click here for file
